# Study on preference and willingness to pay for urban marathon event experience from an embodied perspective

**DOI:** 10.1371/journal.pone.0334308

**Published:** 2025-11-11

**Authors:** Cuixia Yi, Zhipeng Liu, Tao Yang, Kaidi Zhang

**Affiliations:** 1 School of Humanities and Social Science, Xi’an Jiaotong University, Xi’an, Shaanxi, China; 2 Physical Education School, Shaanxi Normal University, Xi’an, Shaanxi, China; 3 School of Economy and Management, Shanghai University of Sport, Shanghai, China; 4 School of Economics and Management, Xi’an Physical Education University, Xi’an, Shaanxi, China; 5 Physical Education School, Guangxi Normal University, Guangxi, China; ISSEP Kef: Universite de Jendouba Institut Superieur du Sport et de l'Education Physique du Kef, TUNISIA

## Abstract

Urban marathons have become important platforms for fulfilling individuals’ aspirations for a better life and promote residents’ health. This study employs a choice experiment method and utilizes embodied theory to explore attributes and design attributes levels of urban marathon products, based on an analysis of social network text content. Through a questionnaire survey of marathon participants, the results reveal that prioritize embodied experiential attributes in the following order: visual experience, auditory experience, extended recollection, self-transcendence, kinesthetic experience, registration fees, and social bonding. Runners with higher incomes and event expenditure prefer visual experiences, while those who participating more frequently exhibit lower preferences for kinesthetic experience. Runners with higher education levels prioritize off-scene experiences. Based on preference heterogeneity, four distinct runner types are identified: ritual-driven runners, audio-visual experience-oriented, achievement-oriented, and social emotion-oriented. Management implications and recommendations for organizers of urban marathon events are provided.

## Introduction

Amid China’s transition to mass tourism [1], and the implementation of national health strategies, sports tourism has emerged as a vital mechanism for advancing public health, stimulating domestic consumption, and restructuring related industries. Urban marathons, with their wide participation and broad socio-economic impact, have become a strategic vehicle for cities to attract visitors and advance sports tourism [2]. For instance, the 2024 Wuxi Marathon attracted more than 260,000 applicants and was estimated to generate ¥ 282 million in local spending across dining, accommodation, transportation, and tourism sectors [3].

By 2025, nearly 70 World Athletics Label road races are expected to be held within China, with the Wuhan Marathon alone receiving over 450,000 registrations—a historic high. However, alongside the rapid growth of marathon events, several challenges have gradually emerged. From the supply side, On one hand, the surge in event supply has resulted in decision fatigue among runners, who often report lacking the physical capacity to participate in multiple events This has further intensified the market polarization, with premier events becoming extremely competitive, while others suffer from mass withdrawals or even cancellation [4]. On the other hand, the exponential increase in participation places immense pressure on event management and services. Some races have lost their accreditation from the Chinese Athletics Association due to organizational shortcomings, such as poor crowd control. From the demand side, as the number of participants and degree of involvement continue to grow, a large number of runners shift from casual runners to serious runners, leading to rising expectations for events [5]. Participants now seek more than just the 42.195-kilometer physical challenge; they look for a comprehensive travel and sports experience that spans the entire pre-, during-, and post-race process [6]. As a form of participatory sports tourism [7], marathon events now place significant emphasis on the runner’s experiential process, which—alongside event quality and service provision—is considered a key component of event attractiveness [8]. At the macro level, participant experience enhances the tourism-related economic impact of events [9] and contributes to destination branding [10]. At the micro level, it influences participants’ motivations, behavioral intentions such as repeat participation, revisitation, and word-of-mouth recommendations [11,12]. Therefore, against the backdrop of an increasingly competitive marathon market, rising participation, and changing expectations, aligning event offerings with runners’ preferences and enhancing participants’ experiences has become a critical issue for the development of urban marathon events. For event organizers, such as event companies and local governments, defining participants’ experience preferences and how much they are willing to pay for these preferences will contribute to the scientific pricing strategy, precise product positioning, and rational resource allocation.

Experience is widely regarded as a subjective construct encompassing emotional, experiential, and symbolic value [13] and is shaped by tourists’ pre-event expectations and the meta-narratives crafted by experience providers [14,15]. Tourists evaluate their experience based on their personal perceptions. Conceptualizing the marathon experience as a product, participants’ preferences and willingness to pay become key to guiding event design and aligning supply with demand. Previous studies revealed that the physical pain induced by distance-running transforms bodily movement into a profound experience, and participants can attain a state of flow and self-transcendence that is rarely achieved through other types of events [16,17]. The quality of such experiences can be evaluated across multiple dimensions, including engagement, challenge, novelty, and enjoyment [18]. However, focusing on participants’ bodily experiences of distance-running itself, existing research has paid relatively little attention to what specific attributes participants prefer most and how much they are willing to pay for their preferences. Additionally, compared to general tourism formats that primarily prioritize sensory experiences, sports tourism places a greater emphasis on body involvement, as its intensity of embodied experience is typically higher [19]. The intrinsic characteristics of urban marathon running further amplify the embodied nature of events tourism, rendering it a particularly salient domain for studying embodied experiential attributes.

Therefore, this study adopts embodied theory to analyze the textual content of urban marathon events on online platforms, aiming to identify participants’ the embodied experiential attributes. Utilizing the text-to-image capabilities of large language models, the study generates visual representations for different attribute levels. A discrete choice experiment is then employed to examine participants’ preferences for various event attributes and to provide recommendations for the design, organization, and management of urban marathon events. The purpose of this study is to investigate (1) what are the specific attributes of urban marathon events under the perspective of embodied theory; (2) what attributes runners prefer most and how much they will to pay; (3) whether any heterogeneity exists in the preference of different runners groups. The novelty of this study mainly includes the following three aspects: First, compared to previous research concerning marathon events, this study examines urban marathon experience attributes based on the textual analysis of social media posts under the embodied perspective. Second, to make the description of attributes easy to be understood, this study builds the choice experiment with the support of text-to-image capabilities of large language models. Third, this research not only examines how demographic characteristics influence participants’ event choices but also explores the heterogeneity of preferences across different participant segments.

## Literature review

### Urban marathon events

Since the modern Olympic Games in 1896, the marathon has been a staple Olympic event. Today, urban marathons often include additional race formats such as half marathons, 10K, and 5K races to encourage broader public participation [20]. As a form of recreational activity, marathon running not only benefits participants by improving physical health, enhancing a sense of belonging, and boosting self-esteem [21] but also plays a significant role in urban economic growth [22]. Existing studies on urban marathons can be generally categorized into three thematic areas: First, research on the development of urban marathons. Zhao, using Hempel’s genetic explanation theory, explored how urban fields, local culture, and participant groups have contributed to the evolution from Olympic marathons to popular urban marathons [23]. Second, research on the economic and cultural impacts of marathon events. Previous research from developed and developing countries has confirmed the positive effect of marathons on the host city’s economic growth [24,25]. Additionally, urban marathons can also contribute to urban cultural development by enhancing civic engagement and promoting an open, inclusive urban image [26]. Similar to findings from developed countries [27], major marathons in cities such as Shanghai and Guangzhou have been shown to strengthen destination branding [10,28]. Third, research on the physical and psychological impacts of marathon participation. Zhang, focusing on local participants, found that marathons influence physical and mental well-being through their material and cultural presence, thereby enhancing participants’ sense of happiness [29].

Overall, the positive impacts of urban marathon events on both participants and destination stakeholders have been widely acknowledged, underscoring the practical significance and value of academic research. The development of urban marathons is influenced by multiple factors, among which participant experience serves as a critical analytical lens and evaluative criterion. Research from the perspective of experience enables a deeper understanding of participant needs and offers more targeted guidance for the planning of events and the sustainable development of host destinations.

### Events experience

Tourism is a purposeful and voluntary movement of people, with its core lying in the production of diverse experiences through spatial displacement, including activities dominated by various content types such as entertainment, emotion, learning, and relaxation [30]. As customers shift their propensity from product and service-oriented to experience-oriented, and positive and memorable experiences can bring customer loyalty, repeat business, and positive word-of-mouth recommendations [31], modern tourism destinations encourage visitors to actively participate in various event activities, thereby constructing experiential systems that include entertainment, education, aesthetic appreciation, and escapism [32]. Consequently, events are increasingly regarded as effective ways for attracting tourists and enhancing destination visibility, with growing scholarly attention on creating customer experience [33,34]. Specifically, studies on marathon event experiences focus on two primary questions: First, what constitutes the marathon experience? As a dynamic process [35], experience enmeshes ongoing interactions between perception, cognition, and emotion [36]. Drawing from Bataille’s concept of inner experience, Ren argues that ultra-marathon runners’ physical suffering equates to a process of acquiring the superego, making physical exertion a profound experience [16]. Zhou proposed an experience threshold model for marathon runners, conceptualizing their experience as a progressive process from anticipation and longing, to release and transcendence, and ultimately to self-breakthrough and personal growth [17]. Yang identified seven perceived dimensions, such as participation, challenge, novelty, and entertainment, for evaluating the quality of marathon experiences through the analysis of online marathon travelogues. Second, what is the relationship between events experience and the tourists-destination relationships? Experience theories suggest that tourists actively pursue experiences aligned with their interests, preferences, and skills [37], while personal, environmental, and interactive factors are considered key drivers of travel experiences [31]. The processual experience of participants is a key factor influencing the attractiveness of marathon events [8]. The attractiveness of international city marathons is closely tied to their tourism effects, necessitating deeper attention to the demand structures shaped by the nature of tourism experiences and emphasizing participants’ perceptions and embodied experiences. Furthermore, existing studies have confirmed the positive impact of sports events experiences on destination brand image, participant satisfaction, and word-of-mouth recommendation intention [10,18].

It is evident that events experience plays a critical role in the interaction between tourists and destinations. However, participants’ evaluations of their experiences are shaped by subjective perceptions and contextual factors, leading to variations in their preferences and willingness to pay. Existing studies primarily focus on defining the nature of the experience and exploring its associations with other factors, while paying limited attention to which specific aspects of the experience participants value most. Research on the preferences and willingness to pay of urban marathon participants can clarify the key attribute of event experience that matter to them, thus providing more specific empirical support for optimizing events experiences, designing tourism products, and formulating pricing strategies.

### Theory of embodiment

The theory of embodiment originates from Maurice Merleau-Ponty’s philosophical notion of the “body-subject,” which initially explored the mind-body relationship from a speculative perspective. The standard theory of cognition assumes that the formation of cognition and knowledge is based on the storage of information that is separated from the sensory modalities through which the information was acquired [38]. While embodied theory considers that cognition and body sensory experience are interwined, and cognitive processes are typically grounded in sensory perceptions, situated action, and bodily states [39,40]. In other words, the core concept of embodiment theory is that our brain is tied heavily to the body in cognitive processes [41], with embodied experiences emerging from the dynamic interaction between individuals and their environments [42]. Since the early 21st century, with the increasing attention given to the interplay between the mind and the body’s physiological structures, action schemas, and spatial interactions, the tourist’s body and its interaction with attractions, environments, and activities have become focal points of tourism studies [43]. Tourism is a strongly embodied experience, as tourists are exposed to unfamiliar sensory stimuli influenced by situational, social, and environmental factors [44]. Consequently, tourists’ experiences are inherently embodied, encompassing proprioception of body position and posture, kinesthetic awareness of limb and muscle movements, and multi-sensory perception through vision, hearing, smell, taste, and touch [45]. Tourists generate a sense of well-being through embodied experiences shaped by sensory input and bodily actions during the visit and continue to construct meaning through embodied recollection and affective memory after the experience ends [46]. In specific contexts such as red tourism sites, embodied engagement enables young visitors to internalize ideological messages through sensory, motor, and semantic stimuli [47]. Existing research on tourism indicates that tourists’ embodied experience arises from the synergistic interaction among the body, the scene, and the interaction between the two [19]. This experience primarily encompasses bodily experiences derived from sensory perception, emotional states shaped by empathic immersion, and embodied interaction with the scene through simulation [45], and can be measured on physical, emotional, and environmental levels [13]. In operationalizing variables from some empirical studies, embodied experience also can be categorized into four dimensions: environmental experience, bodily experience, service experience, and emotional state [48].

Within the embodied framework, sports, grounded in physical activity, are recognized not only for their health benefits but also for their social and experiential value. Previous studies have explored embodiment in sports such as esports and natural based activity [49,50]. These studies emphasize not only visual perception but also the engagement of all senses during physical activity, including auditory, kinesthetic, and proprioceptive perception, focusing on how the body interacts with natural elements through sensory experience, affective responses, and emotions evoked in physical and mindful practices [51]. Through bodily engagement, participants both sense physical feedback and influence future behavior via embodied imagination [52]. Employing embodied approaches to study perception, experience, and culture in sports participation offers a pathway to understanding its sociocultural dimensions and life impacts [53].

Different from general tourism, sport tourism, which is rooted in practices such as play, games, and competition, facilitates higher-level experiential outcomes [19]. In recent years, scholars have utilized embodied to examine sports tourism experience. Through an investigation of 631 visitors to sports tourism destinations, research suggests that the proprioception and kinesthesia in sports tourism activities can affect post-trip behavioral intention [54]. By analyzing materials from online travel notes and on-site interview text, Zhang’s research constructed the story line of the surfing tourism embodied experience, which includes four typical processes of embodied perception, awakening, emotion, and extension [55]. Similarly, research on hiking tourism analyzed travel blogs to investigate the role of embodiment in tourists’ memorable experiences, and physical, social, and emotional embodiment were found to be essential in creating mindful and meaningful experiences [56].

As a typical form of sports tourism, urban marathon events offer experiences that heavily rely on participants’ sensory stimulation, interaction with the environment, and emotional resonance. There has been considerable growth in qualitative and also phenomenological research on the affective and interactional dimensions of embodiment in distance-running [57]. However, existing studies have primarily focused on participants’ bodily experiences of distance-running itself, including themes such as injury, success and achievement, and bodily pain as pleasurable [58]. In contrast, few studies have examined marathons as a form of event tourism product, or explored how such product should be designed to promote participant’s interaction experience and address their emotional needs. Moreover, existing studies have predominantly employed either qualitative or quantitative approaches, often relying on single-disciplinary perspectives rooted in sociology, management, or marketing, thereby leading to insufficient theoretical and methodological integration across disciplines. Since embodiment theory emphasizes the construction of experiential meaning through the interplay of the body, setting, and situational context, its application can provide a clearer and more comprehensive understanding of specific experiential dimensions from the participants’ demand perspective. Since embodiment theory emphasizes the construction of experiential meaning through the interplay of the body, setting, and situational context, its application can offer a clearer and more comprehensive understanding of specific experiential dimensions from the demand perspective.

Overall, research on urban marathon events has reached a relatively mature stage. Although some studies have already addressed participants’ experiences and their positive impacts on the event and host destinations, there remains considerable room for deeper exploration. In terms of research perspective, few studies have employed embodiment theory to examine marathon participation experiences, and the role of the body in sports tourism remains underexplored. Regarding research subjects, limited attention has been given to participants’ experiential preferences and willingness to pay, resulting in a lack of concrete recommendations for events and service designation. Therefore, this study adopts embodiment theory to clarify the experiential attributes of marathon participants and further investigates their preferences and willingness to pay, aiming to inform product innovation and service optimization for urban marathon events.

## Materials and methods

### Research method

This study was conducted by questionnaire and did not involve human clinical trials or animal experiments. According to national laws and institutional guidelines, such research does not require ethics committee approval. This study meets all ethical standards, including the requirement of the supervisor and the institution. All participants were anonymous and volunteered to participate. This study employs a mixed-method research design that integrates structured textual analysis with a choice experiment, aiming to identify event product attributes based on participants’ authentic expressions. Some academics have made specific recommendations for the application of qualitative research methods alongside choice experiments, with a particular focus on identifying attributes and their corresponding levels [59]. For instance, by analyzing online travel agency (OTA) platform comments, a study was designed to examine the attributes of marine wellness tourism products and subsequently investigate tourists’ preferences and willingness to pay [60]. Therefore, according to previous studies and established recommendations for choice experiment design, this research adopts a qualitative-quantitative approach. Specifically, it first conducts a document structure analysis of online discussions to extract attributes that participants genuinely care about, thereby minimizing the subjectivity inherent in researcher-defined attribute selection. Based on the extracted attributes, a choice experiment is then implemented to simulate realistic decision-making scenarios by systematically varying attribute-level combinations. This enables the quantification of individual preferences and provides insights into the underlying trade-offs participants make when evaluating alternative configurations of event characteristics.

#### Document structure analysis.

Document structural analysis has been widely employed in fields such as tourism management and marketing and is commonly adopted to identify product attribute characteristics through a three-level coding process [60]. This approach aims to identify and extract the themes, hierarchies, and logical relationships embedded in document data, facilitating further analysis, synthesis, and interpretation. Based on embodiment theory, this study applies a three-level coding process to analyze online textual content, drawing on the principles of evidence-based analysis to extract concepts and themes, thereby enabling a more comprehensive identification of embodied experiences among marathon participants.

#### Choice experiment.

Choice Experiment (CE) is a method used to investigate individual preferences and decision-making behaviors, widely applied in fields such as marketing, tourism management, and ecological governance [61,62]. By simulating realistic and intuitive choice scenarios, CE allows respondents to make trade-offs among alternative options, thereby inferring their preferences for different attributes [63]. The empirical foundation of CE lies in Random Utility Theory, which posits that consumers make choices based on the relative utility levels of different goods [64]. Accordingly, this study constructs a Random Parameter Logit (RPL) model to empirically analyze marathon participants’ experiential preferences. Under the assumption of utility maximization, the optimal alternative is the one that yields the highest perceived utility for the participant. The utility U_*ji*_ that participant *j* derives from choosing experience option *i* from choice set T is expressed as:


$$U_{ji}=V_{ji}+\varepsilon_{ji}\;$$ (1)

*V*_*ji*_ represents the observable component, indicating participant *j*’s preference for alternative *i,* which is typically modeled as a linear function and can be expressed as:


$$\begin{array}{c} V_{ji}=\sum_{m=\text{1}}^{M}\beta_{m}X_{jim}+\sum_{k=\text{1}}^{K}\gamma_{k}Z_{jk} \end{array}$$ (2)

$X_{ji,m}$ represents the *m*-th attribute level of alternative chosen *i* by participant *j*; $\beta_{m}$ is the coefficient of attribute *m*, reflecting the participant’s preference for that attribute; $Z_{jk}$ denotes the *k*-th individual characteristic of participant *j*; $\gamma_{k}$ is the coefficient of the individual characteristic variable, indicating the influence of personal characteristics on utility.

In Equation (1), *ε*_*ji*_ is the random term representing the unobservable component, which is typically assumed to follow a Type I Extreme Value distribution. According to the utility maximization theory, for any *i* ≠ *n*, when $U_{ji}>U_{jn}$ the probability P*ji* that participant *j* chooses alternative *i* over alternative *n* from the full choice set T is:


$$P_{ji}=P(V_{ji}+\varepsilon_{ji}>V_{jn}+\varepsilon_{jn},\;\forall n\neq i,i\in T,n\in T)$$ (3)

Since *ε*_*ji*_ and *ε*_*jn*_ are random terms that follow the Type I Extreme Value distribution, the probability that participant *j* chooses alternative *i* can be further derived as:


$$P_{ji}=\frac{exp(V_{ji})}{\sum_{n\in T}exp(V_{jn})}$$ (4)

Compared to the Multinomial Logit Model (MNL), the Random Parameter Logit is capable of capturing individual heterogeneity, more accurately describing participants’ choice behavior, accommodating different distributional assumptions, and estimating the effects of various attributes [65]. Therefore, this study adopts the RPL for estimation. The probability that participant *j* chooses alternative *i* from the set of available options T is defined as:


$$P_{ij}=\int \frac{exp(V_{ji}(\beta ))}{\sum_{n\in T}exp(V_{jn}(\beta ))}f(\beta )d\beta ,i\in T,n\in T$$ (5)

Although the RPL is capable of capturing individual-level heterogeneity, it cannot directly identify distinct preference patterns among latent subgroups. To further explore differences in attribute preferences across participant groups, this study employs the Latent Class Model (LCM) for analysis. Latent class analysis is a statistical method used to classify individuals based on their response patterns to observed indicators, thereby identifying group-level heterogeneity [66]. The probability that individual *j* in class S chooses alternative *i* is given by:


$$\begin{array}{c} P_{ji}=\sum_{S}P\left(i|S,X_{ji}\right)R_{jS}=\frac{exp(V_{ji}(S))}{\sum_{n\in T}exp(V_{jn(S)})}\frac{exp(\theta_{s}^{\prime }Z_{j})}{\sum_{S^{\prime }}exp(\theta_{s^{\prime }}^{\prime }Z_{j})} \end{array}$$ (6)

*V*_*ji*_ represents the utility that individual *j* assigns to alternative *i* within latent class *S*; $R_{jS}$ denotes the probability that participant *j* belongs to class *S*; $\theta_{S}$ s the parameter vector for class *S*; *Z*_*j*_ is the feature vector of individual *j*, and *S*’ represents the set of all possible latent classes.

Based on the estimation results of the RPL and LCM models, the marginal willingness to pay (WTP) for a specific attribute change is measured by dividing the estimated coefficient of the marathon event experience product attribute by the estimated coefficient of the price parameter. This helps analyze the relative importance of different product attributes for different categories of participants. The formula is as follows:


$${WTP}_{m}=-\beta_{m}/\beta_{p}$$ (7)

${WTP}_{m\;}$ represents the participant’s willingness to pay for attribute *m*, $\beta_{m}$ is the estimated coefficient for attribute *m*, $\beta_{p}$ is the estimated coefficient for the price attribute.

### Choice experiment process

Firstly, focusing on participants’ choice preferences in marathon events experiences, this study draws on embodied theory and employs discussion texts on social media related to specific marathon events as research data. Through document structure analysis, the study identifies events experiences attributes and levels under the embodied perspective. Secondly, based on the identified attributes, the visualization of marathon experiences attributes is conducted using image-generation functions of two large language models, Gemini and ChatGPT. Finally, SPSS is used to orthogonalize the attribute sets, and a questionnaire is designed accordingly. Data collection is carried out both online and through field surveys.

#### Attributes design and levels setting.

(1) Attributes design

Big data analysis enables the processing of large volumes of user-generated content, such as social media comments and forum discussions, which are highly timely, wide in coverage, and often reflect users’ genuine experiences due to the spontaneous and less manipulated nature of social media feedback. Compared to content from OTA platforms like Ctrip, social platforms such as Rednote and Weibo feature more frequent user posts, larger data volumes, faster updates, and content that tends to express users’ voluntary and authentic opinions. Moreover, these platforms capture participants’ expressions throughout all stages of marathon events—before, during, and after the race.

Given that the field survey of this study is to be conducted in Xi’an, marathon events around the Xi’an region were selected as the retrieval themes to ensure the contextual relevance between respondents and experience attributes. Therefore, Rednote and Weibo were chosen as data sources, and both major events (e.g., “Taiyuan Marathon,” “Chengdu Marathon”) and smaller-scale events (e.g., “Hanzhong Foping Half Marathon”) were set as retrieval keywords. A web crawler was used to collect relevant topics and discussions. After removing invalid entries, a total of 2,279 valid text records were obtained for analysis. The textual data used in this study were collected from publicly accessible sources. All data were anonymized and analyzed in aggregate form. The collection and use of these data complied with the terms and conditions of the respective platforms and were used solely for academic research purposes. No private or personally identifiable information was collected or disclosed.

This study contends that in the context of marathon events, service experience represents an interaction between the body and the scene and thus is regarded as part of scene interaction. Based on the core components and dimensional structure of embodied experience, this study conducts a document structured analysis of online data to extract the experience attributes of marathon events from an embodied perspective. First, open coding is employed to analyze the textual data. Through data fragmentation, comparison, and conceptualization, 29 first-order concepts are extracted. Second, based on these first-order concepts, the internal associations among concepts are examined and aggregated, resulting in 11 second-order themes such as visual experience. Finally, by repeatedly comparing the raw data and drawing on evidence-based analytical methods, the core meanings of the second-order themes are further interpreted and categorized into three overarching dimensions: bodily experience, scene interaction, and emotional state (see Table 1).

**Table 1:** Document structured analysis of marathon events.

Online text^*^	First-order concepts	Second-order themes	Dimensions
I personally felt there weren’t enough scenic views. It would be better if we could run through Minghu Park or Yaoci.	Scenery along the route	Visual experience	Bodily experience
The event used the classic “Hundred-Mile Yellow River Scenic Route,” showcasing landmarks like the Yellow River Mother Statue, Zhongshan Bridge, Yellow River Tower, and Olympic Sports Center.	Cultural landmarks along the route		
When I arrived at the venue, I heard volunteers cheerfully shouting “Welcome to Lanzhou!” The whole experience was filled with uplifting sounds, and everyone greeted each other warmly — it felt like coming home.	Volunteer guidance	Auditory experience	
Starting with 30,000 people and volunteers and citizens cheering along the route made it lively and fun.	Cheering from the audience		
If you’ve trained or are physically strong, a half marathon is manageable. If worried, just follow the pacer.	Encouragement from pacers		
There were sponsor stages along the route with music programs -great vibes.	Musical atmosphere		
Why are there so many snacks on the marathon route? I gained 2 kilos after the race!	Local food	Gustatory experience	
Although Liupanshui is 300m higher than Lanzhou, the cool weather made it much more comfortable.	Race weather	Tactile experience	
I saw Tongchuan at dawn and the sunrise in the morning. I breathed fresh air and felt the vibrant atmosphere.	Fresh air	Olfactory experience	
The route was flat and spacious with reasonable partitions. Although it was crowded, it didn’t feel cramped.	Course design	Kinesthetic experience	
I didn’t find hydration to be a problem. Few supplies in the first 10K is normal — just water is fine.	Physical recovery		
In the actual race: followed plan until 30K, heart rate rose from 31K, body felt worse. At 36K, my HR reached 188; forced myself for 2K, then collapsed.	Pace rhythm		
I only had time to tape under my right knee, but started feeling pain in my left one a few kilometers in. Still finished the full course.	Pain feedback	Proprioceptive experience	
My pace control is okay, but I haven’t done much road running lately. I kept checking pace and adjusting due to bottlenecks. Couldn’t focus fully on pace.	Race focus		
I’ve never experienced such poor service. Weather is nature’s fault, but hotel prices doubled or tripled. Terrible race organization.	Proprioceptive experience	On-site interaction	Scene interaction
Each zone should be bigger. Many runners waited outside check-in areas, causing people to skip check-in.	Service professionalism		
One hour after finishing, full and half marathon runners got personalized AI short videos. Super cool!	Service efficiency		
Free scenic spot visits and transportation for participants-excellent attention to detail.	City friendliness		
The medal is beautiful-the spinning water part is awesome. I kept playing with it on the train ride back.	Tangible memory	Extended recollection	
Lanzhou’s race vest is stylish and practical. I wear it for my morning runs. Saw many people in Taima wearing it too.	Embodied simulation		
The city is clean and spacious, great weather, and the food- delicious, cheap, and abundant. Happiness overload.	City memory		
Long-distance LSD training lacking: missing 25-35K endurance runs led to performance collapse in second half.	Race reflection		
Finally succeeded in the “sub-3:20” plan at Taiyuan Marathon. Congrats to Yong-ge on a big PB and fall race season goal.	Self-transcendence	Self-transcendence	Emotional state
Four years later, I’m back. That young girl is now an auntie- stamina definitely declined.	Self-reflection		
Long-distance LSD training lacking: missing 25-35K endurance runs led to second half collapse.	Experience accumulation		
What gave me a sense of accomplishment was how many out-of-town friends were attracted by my videos and now want to join “Taima” or visit.	Sense of achievement		
A happy day unlocking the new Taima route, and made new friends too.	Expanding social network	Social bonding	
First time in Taiyuan-food and sightseeing spree with good friends again.	Maintaining social relationships		
Running the Binzhou Half this Saturday with my running buddy and pacer.	Strengthening runner bonds		

*The original text is written in Chinese.

In the results of the document structured analysis, themes with higher frequencies indicate stronger perceptions among participants. Given the operational limitations of choice experiment, according to previous studies [60], this study selects the six most frequently mentioned second-order themes within the three dimensions of embodied experience as the core attributes of marathon embodied experience, including visual experience, auditory experience, extended recollection, self-transcendence, kinesthetic experience, and social bonding. Specifically, the visual experience attribute refers to the scenery perceived by participants through their visual senses during the marathon, including both natural and cultural landscapes. It serves as a critical element for constructing a destination image. Aesthetic course environments and iconic landmarks along the route can influence the behavioral intentions of sports tourism participants [28]. The auditory experience attribute refers to the sensory experience acquired through hearing during the marathon. Interactions with volunteers, cheers from spectators, and encouragement from pacers all serve to stimulate participants, enhance their confidence, and help them overcome difficulties encountered during the race. The kinesthetic experience attribute primarily involves the runner’s perception of their bodily state during the competition, including muscle load, pace control, and physical coordination. Course design—such as width, slope, and turnaround points—affects the runner’s rhythm and physical load, while the quality of on-course supplies influences running performance and recovery, especially in the latter stages of the race. The extended recollection attribute reflects participants’ post-race interactions with the event, including race reviews and the possession of tangible items such as medals and T-shirts. These contribute to the formation of scenario-based memories, allowing participants to reshape their race experience through memory even after the event has ended. The self-transcendence attribute refers to psychological growth and cognitive breakthroughs participants experience after engaging with the marathon environment, which involves a deeper exploration of one’s value and potential, eventually transforming into an emotional state. The social bonding attribute highlights interpersonal interaction and emotional connections during the race. These include forming new relationships, strengthening bonds with fellow runners, and reinforcing existing friendships through running—thereby enriching the emotional dimensions of the marathon experience.

(2) Levels setting

Based on the document structure analysis of online textual data, the study identified six attributes of urban marathon events from an embodied perspective. To ensure sufficient variation for preference elicitation while maintaining plausibility and relevance to potential participants, the attribute levels were informed by the results of existing literature, content of online discussions, prevailing industry practices, and the need for choice experimental design. Specifically, following established research on the choice experiments [60,61], each attribute was specified with three distinct levels to enable a more refined simulation and assessment of participants’ preferences. To enhance contextual realism, each level was embedded in concrete elements of the event. For instance, the levels of the visual experience attribute were operationalized by varying the types of landscapes encountered along the racecourse [28]. Drawing on both online discussions and prevailing industry practices, two trends were observed: first, participants frequently post photos highlighting impressive natural scenery or distinctive cultural landmarks; second, event organizers often design race routes that incorporate local characteristics to showcase regional identity and cultural uniqueness, such as Lanzhou marathon whose racecourse was designed along the Yellow River [67].

Consequently, this study distinguishes the visual experience attribute through different types of race course landscape: ordinary city roads represent the basic level; city roads with lush vegetation and landmark buildings represent the intermediate level; and impressive, breathtaking landscapes constitute the advanced level. The remaining five attributes were defined in a similar manner, with three contextually grounded levels designed to reflect realistic variations in event features. For the auditory experience attribute, a course with cheering spectators along some sections serves as the basic level; a full course with large, enthusiastic crowds serves as the intermediate level; and the addition of pre- and post-race music ambiance defines the advanced level. In terms of kinesthetic experience attribute, the study differentiates based on the quality of post-race recovery support—basic sports refreshments such as bottled water and electrolytes define the basic level; the inclusion of energy gels and medical spray constitutes the intermediate level; and the provision of sufficient stretching and recovery assistance defines the advanced level. For the extended recollection attribute, tangible products that evoke participant memories are used as key elements, with different standards of medal and race pack design used to set three levels. Regarding self-transcendence, the attribute is defined by runners’ pursuit of identity and achievement. The levels are represented by combinations of personal best medals, multi-year participation awards, and personalized bib numbers. For the social bonding attribute, the element is the social convenience provided by the event. The attribute levels are set through a combination of online runner-matching activities, team registration options, and distribution of “find-a-friend” coupons. To ensure the robustness and reliability of the WTP results, the study sets four price levels—RMB 100(≈USD 14/€12), 150 (≈USD 21/€18), 200 (≈USD 28/€24), and 249 (≈USD 35/€30)—based on current marathon registration fees [68]. Each attribute and its corresponding levels were visually represented using a large language model’s text-to-image generation function, specifically Google Gemini 1.5 and OpenAI ChatGPT-4o. Before the formal survey, a pilot test with 20 participants was conducted to assess the interpretability and validity of the visual materials. Runners were invited to comment on the clarity and contextual relevance of the images. The majority of participants (over 80%) reported that the images accurately reflected the levels attributes, and according to the remaining feedback, we made minor adjustments to improve clarity and contextual accuracy. The results are shown in Table 2, and the images are provided as Supplementary Material (S2 File).

**Table 2:** Attributes levels setting.

Attributes	Levels and description		
	1	2	3
Visual experience (VIS)	Ordinary city roads	City roads with lush vegetation and landmark buildings	Impressive, breathtaking landscapes
Auditory experience (AUD)	Cheering spectators along some sections	Full course with large, enthusiastic crowds	Full course with large, enthusiastic crowds, pre-/post-race music ambiance
Kinesthetic experience (KIN)	Basic sports refreshments bottled water and electrolytes	Bottled water and electrolytes; and energy gels and medical spray	Bottled water and electrolytes; energy gels and medical spray; sufficient stretching and recovery assistance
Extended recollection (ERE)	Finisher medals and participant T-shirts with distinctive local characteristics	Finely designed medals with local cultural features, and participant T-shirts/finisher commemorative shirts	Personalized medals with the participant’s name engraved, along with participant T-shirts/finisher shirts/water bottles
Self-transcendence (STR)	Personal best medals	Personal best medal, multi-year participation awards	Personal best medal, multi-year participation awards, and personalized bib numbers
Social bonding (SBO)	Online runner-matching activities	Online runner-matching activities; team registration options	Online runner-matching activities; team registration options, and distribution of “find-a-friend” coupons

#### Choice set and questionnaires.

In the discrete choice experiment designed in this study, six three-level experience attributes and one four-level price attribute were included. According to the full factorial design, random combinations of different attributes and levels would result in a total of 36 × 4 = 2,916 possible combinations, which would inevitably lead to decision-making difficulties for respondents [69]. Therefore, an orthogonal experimental design was conducted using SPSS to generate 18 product choice sets, with 3 unreasonable combinations subsequently removed. The remaining 15 choice sets were organized into 5 choice scenarios, each containing 3 product alternatives. Respondents were asked to make selections within these 5 scenarios.

#### Location site and data collection.

This study selected Xi’an, China, as the survey area. As a major city in northwest China with a resident population of 13 million, Xi’an hosts well-known events such as the Xi’an Marathon (gold competition subgroup, World Athletics) and the Xi’an City Wall International Marathon, fostering a strong running culture. In addition, as a key transportation hub in western China, its convenient transport links enable local runners to travel easily to surrounding cities such as Lanzhou, Chengdu, and Taiyuan to participate in various marathon events. The survey was conducted in two phases. In the first phase, convenience sampling was used to invite runners to complete questionnaires at the finish lines of the half and full marathons of the 2024 Xi’an Marathon (November 3, 2024). For serious runners, WeChat contact information was collected solely to facilitate subsequent snowball sampling. In the second phase (November 4–25, 2024), electronic questionnaires were distributed via WeChat running club groups through the networks of these runners. A total of 373 questionnaires were distributed, and 335 valid responses were retained after excluding those with missing answers, inattentive completion, or patterned responses (S1 Table). The final sample size exceeds the minimum requirement calculated using Orme’s empirical rule for conjoint analysis [62,70] and meets the recommended thresholds for mixed logit model estimation. All participants were fully informed about the purpose and content of the study prior to their participation. Participation was voluntary, and all responses were collected anonymously. Sample characteristics and descriptive statistics are shown in Table 3.

**Table 3:** Profile of respondents (n = 335).

Characteristic	Category	%	Characteristic	Category	%
**Gender(gen)**	Male	76.4	**Running group (group)**	Yes	62.7
	Female	23.6		No	37.3
**Age(age)**	18 or younger	1.5	**Running career (career)**	≤1 year	26.3
	18–25	18.2		1–3 years	33.7
	26–35	23		3–5 years	17
	36–45	31		5–7 years	9.6
	46–55	20.6		≥7 years	13.4
	56–65	4.2	**Events participate annually (events)**	1–3 events	56.1
	≥65	1.5		4–6 events	29.6
**Education(edu)**	Middle school or less	2.7		7–9 events	8.7
	High school	10.4		≥10 events	5.7
	Vocational college	26.9	**Events participate (par)**	Full marathon	30.7
	Undergraduate	41.8		Half marathon	52.2
	Postgraduate	18.2		Fun run (5–10 km)	17
**Profession**	Student	19.1	**Cost on equipment per year(¥** ^*^ **) (equ)**	≤¥1000	36.7
	Civil servant	23.3		¥1001–2000	31.6
	Enterprise employee	26.6		¥2001–3000	15.8
	Educational technician	6.6		¥3001–4000	5.7
	Freelancer	4.5		≥¥4001	10.1
	Private owner	11.0	**Cost on per events (¥** ^*^ **) (cost)**	≤¥500	23.9
	Farmer	0.3		¥501–700	13.4
	Retiree	4.2		¥701–1000	21.8
	Other	4.5		¥1001–1500	19.4
				≥¥1500	21.5
**Income (¥** ^*^ **) (income)**	≤¥3000	22.4			
	¥3001–5000	19.4			
	¥5001–8000	28.1			
	¥8001–10,000	13.4			
	≥¥10,001	16.7			

*¥ 1 ≈ USD 0.14 or € 0.126.

According to the 2024 China Road Running Races Blue Book [71], a total of 749 road running events were held across China in 2024, including 254 full marathons (32%) and 442 half marathons (56%). Among all finishers, 77% were male and 23% female. Runners aged 35–45 accounted for 37% of all participants. These proportions are broadly consistent with the demographic profile of our sample, suggesting a certain degree of representativeness in terms of gender, age structure, and event participation types.

## Results

The model parameters were estimated using Stata 18 software. The estimation was based on a sample size of 5,025 observations (335 respondents × 5 choice sets × 3 alternatives).

### Analysis of participant preferences for urban marathon events attributes

The study first employed RPL for estimation. Based on existing literature and established practice [72,73], the price attribute—registration fee (PAY)—was specified as a fixed parameter, while the six experiential attributes, such as visual experience (VIS), were modeled as random parameters following a normal distribution. As shown in Table 4, both the fixed and random parameters are statistically significant at the 1% level. The coefficients of visual experience (VIS), auditory experience (AUD), kinesthetic experience (KIN), extended recollection (ERE), and self-transcendence (STR) are positive and consistent with expectations. This indicates that as the levels of these five attributes improve, participants derive greater experiential utility and are more inclined to choose event products with higher levels of these attributes. Among them, the coefficients suggest that participants exhibit stronger preferences for visual experience, auditory experience, and self-transcendence. The coefficient of registration fee (PAY) is negative and significant, implying that participants’ willingness to participate decreases as registration costs increase. Interestingly, the coefficient of social bonding (SBO) is also negative and significant, suggesting that, in the general sample, participants are less likely to choose options with higher levels of this attribute.

**Table 4:** Results of participant preferences for urban marathon events.

Variable	Parameter Estimates (model 1)	
	Coef.	SE
**VIS**	2.558***	0.559
**AUD**	2.439***	0.469
**KIN**	0.442***	0.123
**ERE**	0.508***	0.092
**STR**	1.196***	0.290
**SBO**	−1.913***	0.477
**PAY**	−1.144***	0.211
Standard Deviation of Random Parameters		
**sd(VIS)**	1.164	0.116
**sd(AUD)**	1.050	0.094
**sd(KIN)**	0.274	0.101
**sd(ERE)**	0.134	0.229
**sd(STR)**	0.003	0.088
**sd(SBO)**	0.004	0.146
**Obs**	5025	
**Prob > chi2**	0.000	
**Wald chi2**	80.55	
**Log likelihood**	−1672.633	

*p < 0.1, **p < 0.05, ***p < 0.01.

The standard deviations of the random parameters are also statistically significant. The relatively large standard deviations for visual experience (VIS), auditory experience (AUD), kinesthetic experience (KIN), and extended recollection (ERE) suggest substantial heterogeneity in preferences across individuals for these attributes. By contrast, the standard deviations for social bonding (SBO) and self-transcendence (STR) are close to zero, indicating limited heterogeneity in preferences for these attributes.

### Heterogeneity analysis of participant preferences for marathon events attributes

#### Heterogeneity analysis based on interaction terms with runner characteristics.

The results of the standard deviations of the random parameters in Model 1 indicate the presence of preference heterogeneity among runners for the four attributes. Therefore, this study further incorporates specific characteristics, such as gender, age, cost on equipment per year (equ), education level and events participate per year (events), into Model 1 to identify preference differences across subgroups of participants. The estimation results are presented in Table 5. In Model 2, the interaction terms between visual experience (VIS) and age, income, and average race spending are statistically significant. Specifically, the interaction coefficients for income and race spending are positive, indicating that runners with higher income and greater average race expenditures are more likely to prefer courses with scenic landscapes and visually striking experiences. In contrast, the negative coefficient for age suggests that older participants tend to place less importance on visual experience. In Model 3, the interaction between auditory experience (AUD) and education level is significantly negative, implying that individuals with higher educational attainment place less emphasis on auditory aspects such as event atmosphere. In Model 4, the interaction between kinesthetic experience (KIN) and the number of races participated in annually is significantly negative, suggesting that runners who frequently participate in races are less concerned with kinesthetic aspects such as refreshment stations, possibly focusing more on the race content and challenge itself. In Model 5, the interaction between extended recollection (ERE) experience and education level is significantly positive, indicating that extended recollection elements such as medals and cultural souvenirs are more appealing to individuals with higher levels of education.

**Table 5:** Results of heterogeneity analysis based on interaction terms with runner characteristics.

Variable	Model 2		Model 3		Model 4		Model 5	
	Coef.	SE	Coef.	SE	Coef.	SE	Coef.	SE
**PAY**	−1.117^***^	0.210	−1.140^***^	0.211	−1.159^***^	0.213	−1.173	0.212
**Attributes**								
**VIS**	2.042^**^	0.767	2.549^***^	0.558	2.591^***^	0.562	2.663	0.562
**AUD**	2.395^***^	0.467	3.290^***^	0.729	2.467^***^	0.472	2.497	0.472
**KIN**	0.438^***^	0.123	0.447^***^	0.123	0.171^***^	0.339	0.454	0.123
**ERE**	0.506^***^	0.092	0.505^***^	0.092	0.509^***^	0.093	−0.109	0.369
**STR**	1.168^***^	0.289	1.200^***^	0.290	1.210^***^	0.292	1.236	0.292
**SBO**	−1.869^***^	0.474	−1.915^***^	0.476	−1.937^***^	0.479	−1.992	0.480
**Standard Deviation of Random Parameters**								
**sd(VIS)**	1.051	0.111	1.1157	0.1146	1.1576	0.1164	1.105	0.114
**sd(AUD)**	1.025	0.093	0.9908	0.0917	1.0430	0.0942	1.013	0.094
**sd(KIN)**	0.273	0.100	0.2666	0.1015	0.2183	0.1224	0.343	0.090
**sd(ERE)**	0.091	0.270	0.0801	0.2987	0.2357	0.1486	0.021	0.321
**sd(STR)**	0.002	0.087	0.0022	0.0870	0.0036	0.0907	0.002	0.094
**sd(SBO)**	0.010	0.159	0.0082	0.1575	0.0084	0.1512	0.004	0.150
**Interaction term**	VIS		AUD		KIN		ERE	
**gen**	−0.087	0.200	−0.152	0.177	0.009	0.103	0.054	0.116
**age**	−0.182^**^	0.081	0.111	0.072	0.050	0.042	−0.073	0.047
**edu**	0.079	0.092	−0.191^**^	0.082	0.024	0.048	0.137^**^	0.054
**income**	0.234^***^	0.076	−0.071	0.067	−0.010	0.039	0.062	0.044
**equ**	0.051	0.073	−0.028	0.065	0.025	0.038	0.049	0.042
**career**	−0.049	0.074	−0.057	0.066	0.025	0.038	−0.027	0.043
**par**	0.041	0.147	0.144	0.130	−0.058	0.076	−0.038	0.085
**events**	−0.096	0.106	0.039	0.094	−0.109^**^	0.055	−0.039	0.062
**cost**	0.214^***^	0.072	−0.098	0.064	0.065	0.037	0.072^*^	0.042
**Prob>chi2**	0.000		0.000		0.000		0.000	
**Waldchi2**	104.71		94.18		92.93		100.36	
**Loglikelihood**	−1653.9666		−1661.1346		−1664.5629		−1660.5179	

*p < 0.1, **p < 0.05, ***p < 0.01.

#### Heterogeneity analysis based on latent class of preference.

Although the introduction of interaction terms in the baseline model helps reveal the moderating effect of individual characteristics on race attribute preferences, which partially reflects preference heterogeneity, it still fails to account for the impact of latent unobserved factors such as cognitive styles and personal motivations on preferences. Therefore, the study employs the Latent Class Model (LCM) to further identify latent preference groups and explore the heterogeneity in race attribute preferences across different groups. Before utilizing the LCM model, the number of classes needs to be determined through AIC (Akaike Information Criterion) and BIC (Bayesian Information Criterion). The model with smaller values of AIC and BIC fits better. When the two criteria suggest different numbers of classes, BIC is prioritized [72]. The results show that although AIC continuously decreases from 3 to 5 classes, BIC reaches its minimum at 4 classes and then increases sharply after 5 classes (Table 6). Therefore, the study selects 4 classes as the optimal number of classes.

**Table 6:** Criteria for selecting the number of latent classes.

Class	Log Likelihood	AIC	BIC
**2**	−1636.5148	3303.03	3360.24
**3**	−1587.1953	3220.39	3308.12
**4**	−1564.359	3190.72	3308.96
**5**	−1547.7935	3173.59	3322.34
**6**	−1531.946	3157.89	3337.16
**7**	−1528.20	3166.41	3376.18

The results of the LCM analysis are shown in Table 7. Based on the size of the coefficients for each attribute, the latent classes are defined as follows: ritual-driven, audiovisual experience-driven, achievement-oriented, and emotional social-driven. Each group accounts for 10.5%, 39.5%, 29.4%, and 17.6% of the total sample, respectively. Runners in ritual-driven group emphasize on the commemorative value of the race, focusing on attributes like medals, race shirts, and finisher shirts. They do not prioritize sensory experiences, such as visual, auditory, and kinesthetic experiences, during the event. Runners in audiovisual experience-driven group have a stronger preference for the visual and auditory aspects of the race, such as the scenery along the course and the atmosphere at the event. They are less sensitive to the registration fee. Apart from having a positive preference for audiovisual experiences, runners in achievement-oriented are more focused on self-actualization, valuing the atmosphere and sense of self-improvement created by the event. They tend to avoid social interaction and kinesthetic experiences, suggesting that these runners prefer to experience immersion and self-fulfillment through independent participation. Runners in emotional social-driven group show a significant positive preference for social emotional attributes and have a positive attitude toward the kinesthetic experiences and extended recollection. They are less sensitive to price, indicating that they are more inclined to use the race as a means of building and maintaining emotional connections with others. They value the event’s physical experiences and cultural identity, willing to pay a higher registration fee for a more enriched experience.

**Table 7:** Results of latent class model analysis.

Attributes	Class 1	Class2	Class 3	Class 4
	Ritual-driven	Audiovisual experience-driven	Achievement-oriented	Emotional social-driven
**PAY**	−0.075***	−0.004*	0.009	1.653***
**VIS**	−3.621***	0.871***	2.062**	−5.657***
**AUD**	−6.685***	0.337*	4.588***	−3.207***
**KIN**	−3.08***	0.17	−3.83***	2.887***
**ERE**	2.027***	0.085	−1.304*	1.919***
**STR**	−0.235	0.116	1.503***	−3.429***
**SBO**	−0.337	−0.331	–	5.922***
**sample ratio**	0.105	0.395	0.294	0.206

*p < 0.1, **p < 0.05, ***p < 0.01; for Class 3, the standard error and p-value of the SBO variable could not be estimated due to complete separation.

### Analysis of WTP for marathon events attributes based on participant preferences

Based on the results from the latent class model, the study calculates the marginal willingness to pay for different attributes by each latent group using the estimated coefficients for each attribute and the registration fee (Table 8). Ritual-driven runners have the highest willingness to pay for the extended recollection (¥ 27). Audiovisual experience-driven runners have the highest willingness to pay for visual and auditory experiences, with values of ¥ 216.8 and ¥ 83.8, respectively, which are significantly higher than for other attributes. The coefficients for the registration fee attribute of achievement-oriented and emotional social-driven groups are positive.

**Table 8:** Results of latent preference classes’ willingness to pay.

Attributes	Class 1	Class2	Class 3	Class 4
	Ritual-driven	Audiovisual experience-driven	Achievement-oriented	Emotional social-driven
**VIS**	−48.3	216.8	229.7	−172.8
**AUD**	−89.6	83.8	510.8	−97.8
**KIN**	−41.6	42.8	−426.4	88.1
**ERE**	27.0	21.5	−145.1	58.3
**STR**	−3.1	28.3	167.6	−104.4
**SBO**	−5.0	−82.0	–	180.8

## Discussion

**Fig 1:**
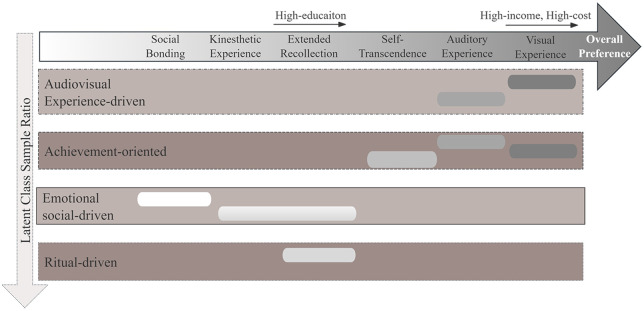
Overview of Attribute Preferences: overall rankings, demographic variations, and latent class segmentation.

This study explored urban marathon participants’ preferences for embodied experiential attributes. As illustrated in Fig 1, the findings indicate notable differences in attribute preferences, demographic heterogeneity, and distinct latent preference classes among runners.

Results from the RPL indicate that the coefficients for visual experience (VIS), auditory experience (AUD), kinesthetic experience (KIN), extended recollection (ERE), and self-transcendence (STR) are all positive, suggesting these attributes significantly enhance runners’ choice probability. Consistent with previous research on long-distance running, visual (VIS) and auditory experience (AUD) are valued by most participants [74,75], followed by self-transcendence (STR). With marathons evolving from competitive events to mass participatory festivals [23], urban marathons have become celebratory in nature and a form of tourism. Participants increasingly perceive races as opportunities to explore cities, and visually captivating race environments significantly influence their preferences [28]. Interaction effect analysis further shows that high-income and high-spending participants are more inclined to choose events with enhanced visual features. Prior studies have noted that income levels strongly shape individuals’ consumption structures and leisure preferences [76,77]. High-income runners, as cultural consumers with aesthetic consciousness [78], tend to seek immersive experiences that blend sports with leisure [79], exhibiting stronger preferences and a higher willingness to pay for superior visual race settings. In addition, auditory stimuli such as warm-up and cool-down music, as well as cheering from enthusiastic spectators along the course, are also key sources of perceived value for runners. For example, the Lanzhou Marathon—renowned for its atmosphere—fully upgraded its “Cheering Support” activity in 2025, injecting new energy into the event [80]. Self-transcendence (STR) also demonstrates a strong positive effect on runners’ preferences, particularly among the “achievement-oriented” group, for whom it stands out as an important attribute beyond visual and auditory experience. Previous research indicates that marathon runners tend to reinterpret physical fatigue through a sense of meaning and accomplishment, extending positive emotional experiences by showcasing their athletic achievements [81]. Whether an event can facilitate self-overcoming and experiential growth plays a crucial role in shaping runners’ embodied affective experiences.

Kinesthetic experience (KIN) and extended recollection (ERE) also exhibit positive preference coefficients, indicating that runners value both the physical sensations during the race and the emotional recollections after finishing. Kinesthetic perception constitutes a unique experiential dimension that distinguishes sports tourism from other forms of tourism [19]. In city marathons, particularly full marathons, amateur runners often encounter intense physical exhaustion and difficulty maintaining pace, commonly referred to as “hitting the wall” [82]. In this context, sufficient medical support and nutritional supplies in the later stages of the race can enhance physical performance, while post-race recovery services (e.g., stretching) can further optimize the overall kinesthetic experience [83]. Runners’ preference for kinesthetic experience reflects their growing emphasis on physical self-management and the broader trend toward more professional and scientific participation [84]. However, the negative and significant interaction between kinesthetic experience and events participation annually indicates notable heterogeneity between core runners and casual participants. Casual participants place greater emphasis on kinesthetic aspects (e.g., refreshment stations). In contrast, core runners, consistent with Serious Leisure attributes such as perseverance, career commitment, and strong identification [85], rely less on physical support and focus more on intrinsic challenges and symbolic outcomes. This divergence is most evident in kinesthetic experience, while preferences for other attributes remain largely aligned.

Preferences for extended recollection experiences (ERE) indicate that commemorative elements such as medals, race shirts, and finisher gear can extend emotional engagement with the event [86]. Heterogeneity analysis reveals a significant positive correlation between this attribute and education level, suggesting that runners with higher education levels are more inclined toward symbolic and ritualistic elements of the event, particularly those embodied in tangible souvenirs such as medals and cultural merchandise. On the one hand, this may reflect a greater likelihood of participation in sports events and travel among highly educated individuals [87]; on the other, it may relate to their stronger capacity and tendency for self-expression [88], enabling them to convert embodied experiences into shareable social capital through symbolic objects.

Runners’ preferences for social bonding (SBO) exhibit considerable variation. On the one hand, the negative coefficient for this attribute in the overall model suggests that most participants prioritize the intrinsic experience of the race itself, rather than viewing the event as a means to initiate or maintain social relationships. This may be due to the perception that social interactions and networking require additional time and energy investments, thereby increasing some participants’ “implicit costs” and reducing their willingness to select such options. For instance, many recreational runners are accustomed to running alone during regular training due to mismatched pacing with others [81], On the other hand, the emotional social-driven group demonstrates a strong preference for this attribute, indicating that social emotional bonding constitutes a key source of event attractiveness for this segment. Therefore, while the overall impact of this attribute appears limited, it holds significant value for specific subgroups of runners.

The latent class model reveals that the “audiovisual experience-driven” group accounts for the largest proportion (39.5%) of all latent classes. This group also exhibits the highest willingness to pay for visual (¥216.8) and auditory (¥83.8) experiences, far exceeding other groups. This finding implies that nearly 40% of runners regard on-site visual and auditory experiences as key determinants in their race participation decisions. Additionally, although “ritual-driven” runners are more price-sensitive, they are also more willing to pay for ritualized experiences embedded in off-site interactions, further highlighting the importance of this attribute in shaping emotional connections with the event. However, for the “achievement-oriented” and “emotional social-driven” groups, the coefficients for the registration fee attribute are positive, which contradicts the economic assumption that “an increase in price decreases utility.” [89] This could be due to these groups being less sensitive to price or other underlying motivations. For example, during the actual survey, some runners mentioned feedback such as “I always choose the most expensive one because most of the event services must be the most valuable.” The preferences of “emotional social-driven” runners for social bonding, and those of “achievement-oriented” runners for Self-transcendence, suggest that these participants value not only the functional returns of the event, such as physical challenge or competitive performance, but also regard the race as a vehicle for realizing personal meaning or emotional belonging [90]. Consequently, they tend to prioritize the symbolic significance embodied in the event over its economic cost. From the perspective of symbolic consumption theory [91], a higher price is no longer perceived merely as a financial burden, but as a bearer of social meaning and personal identity [92]. In this context, price functions as a symbolic signal of value and self-concept, making these individuals more inclined to choose higher-priced options that align with their aspirational identity or desired social image [93].

## Conclusion and implication

### Conclusion

This study, grounded in embodied experience theory and informed by document structure analysis, employs a discrete choice experiment (DCE) to identify runners’ preference attributes in the context of urban marathon event experiences. Visualization of different attribute levels was achieved through large language models’ text-to-image capabilities. A random parameter logit and latent class model were utilized to capture runners’ preferences and heterogeneity. Conclusions are as follows: First, participants’ preferences for urban marathon experience attributes, in descending order of importance, are: visual experience, auditory experience, extended recollection, self-transcendence, kinesthetic experience, registration fee, and socio-emotional bonding. Among these, registration fees and socio-emotional bonding exerted a negative effect on choice in the overall model. Second, significant heterogeneity exists in runners’ preferences for four attributes: visual experience, auditory experience, extended recollection, and self-transcendence. In terms of individual characteristics heterogeneity, runners with higher income and event expenditure show stronger preferences for visual experiences; those who participate more frequently in races exhibit lower preferences for kinesthetic experience; and more highly educated runners place greater emphasis on extended recollection. Third, participants can be classified into four groups based on their preferences: “ritual-driven” runners are more price-sensitive but value extended recollection. “Audiovisual experience-driven” runners, the largest group in the sample, show strong preferences for visual and auditory aspects of the event and have the highest willingness to pay. “Achievement-oriented” runners, in addition to valuing audiovisual experiences, are inclined toward events that offer self-transcendence attributes. “Emotional social-driven” runners are not price-sensitive and show strong preferences for social bonding

### Implication

To address the supply-demand mismatch in current urban marathon events, from a management perspective, event organizers should focus on accurately identifying runner needs and optimizing the structure of event offerings. On the one hand, relevant administrative organizations and event companies need to jointly establish a shared marathon event database through formal agreements to break down “data silos” and fully utilize existing data resources. On the other hand, during event planning, organizers should consider the local conditions and event level, and leverage AI technologies to analyze potential participant profiles and identify the heterogeneous preferences of runners. Based on this, differentiated products and operational strategies can be formulated for various market segments, thereby achieving a more diverse and targeted supply to meet the growing individualized demands of runners better.

In terms of event product design, organizers need to prioritize enhancing participants’ embodied experience through innovative offerings. Specifically, first, iconic city landmarks and local scenic features should be fully leveraged to create visually appealing routes. Immersive audiovisual atmospheres can be cultivated through scene design, audience interaction, and cheering activities along the course. For instance, the Lanzhou Marathon follows the Yellow River and connects with landmarks such as the Yellow River Tower, while coordinating with various stakeholders to build a cheering network that significantly enhances visual engagement and social interaction, thereby improving the event’s overall reputation. Second, organizers should improve runners’ physical experience, especially for novice and recreational participants, by optimizing race-day provisions, ensuring comprehensive medical emergency coverage, and offering post-race services such as guided stretching and icing. For events with more experience and a stable participant base, enhancing service quality can improve the physical sensations of running and better accommodate the needs of entry-level participants. Third, organizers need to focus on the design and innovation of core symbolic items such as finisher medals, race souvenirs, and finisher apparel. On one hand, incorporating local cultural elements can strengthen the event’s sense of place; on the other hand, personalized mechanisms, such as digital medals, achievement displays, and birthday-number bibs, can enhance participants’ sense of ceremony and emotional identification. For instance, the 2025 Wuxi Marathon first introduced a “digital medal” system, enabling each runner to access personalized records and commemorative content through identity-linked activation. Lastly, organizers may introduce initiatives such as group registration for running clubs, social-style aid stations, and post-race social activities to meet runners’ emotional and social bonding needs. These efforts can strengthen group cohesion and cultural resonance. For example, the 2025 Suzhou Marathon launched a “Sub-3 Running Club Elite Race” with substantial prize money to encourage group participation and collective engagement.

### Limitations

The present study still has several limitations. First, this study primarily examined preferences through convenience and snowball sampling of urban marathon participants in Xi’an, which may limit the generalizability of the findings. Future research could enhance external validity by employing larger, randomly selected samples and conducting international comparisons. Second, in terms of research methodology, due to the practical constraints associated with the implementation of the choice experiment, this study examined only six attributes of urban marathon events. Future research could incorporate a broader range of attributes and levels to capture participants’ preferences more comprehensively. Third, concerning the research tool of AI-generated images, on the one hand, the transformation from textual descriptions to visual representations may introduce ambiguity, potentially leading to misinterpretation of event attributes. On the other hand, the AI-generated images offer only a two-dimensional portrayal, which may be insufficient to convey the multi-dimensional and immersive nature of real-world event experiences. Future research could utilize a longitudinal design and incorporate multi-modal generative tools, such as text-to-video or text-to-3D simulations, to mitigate narrative ambiguity and enhance the experiential realism of event representations. Lastly, this study quantified certain subjective experiences into product attributes to ensure feasibility and consistency in the choice experiment, which may have simplified their complexity. Future research could employ complementary qualitative or mixed-method approaches to capture the richer, multi-dimensional nature of embodied experiences.

## Supporting information

S1 Table All used data. This table contains the data used tin the article.
(XLSX)

S2 File Text-to-image data. This file contains all images generated for the attribute levels described in Table 2.
(ZIP)
