# Supplementary figures and images for: Study on preference and willingness to pay for urban marathon event experience from an embodied perspective
Source: PLoS One. 2025 Nov 11;20(11):e0334308. doi: 10.1371/journal.pone.0334308 (PMC12604802; doi:10.1371/journal.pone.0334308)

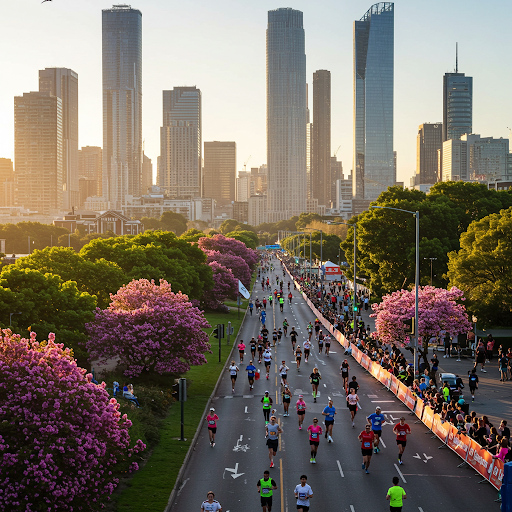

Supplement: S2 File — This file contains all images generated for the attribute levels described in Table 2. (ZIP) [file pone.0334308.s002.zip › S2_File/1-VIS 1.png]

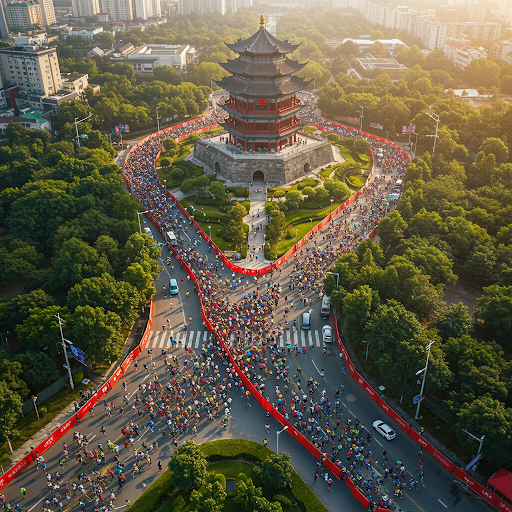

Supplement: S2 File — This file contains all images generated for the attribute levels described in Table 2. (ZIP) [file pone.0334308.s002.zip › S2_File/1-VIS 2.png]

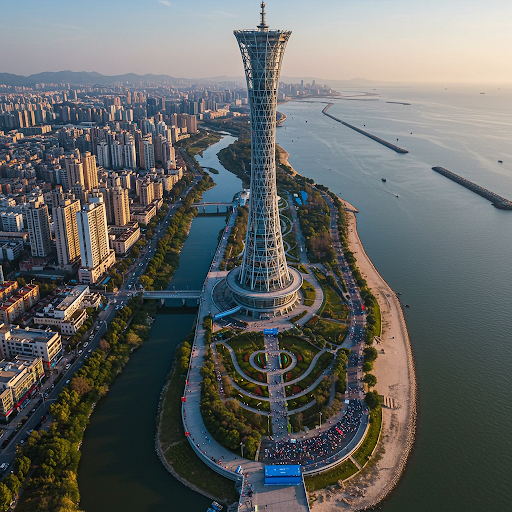

Supplement: S2 File — This file contains all images generated for the attribute levels described in Table 2. (ZIP) [file pone.0334308.s002.zip › S2_File/1-VIS 3.png]

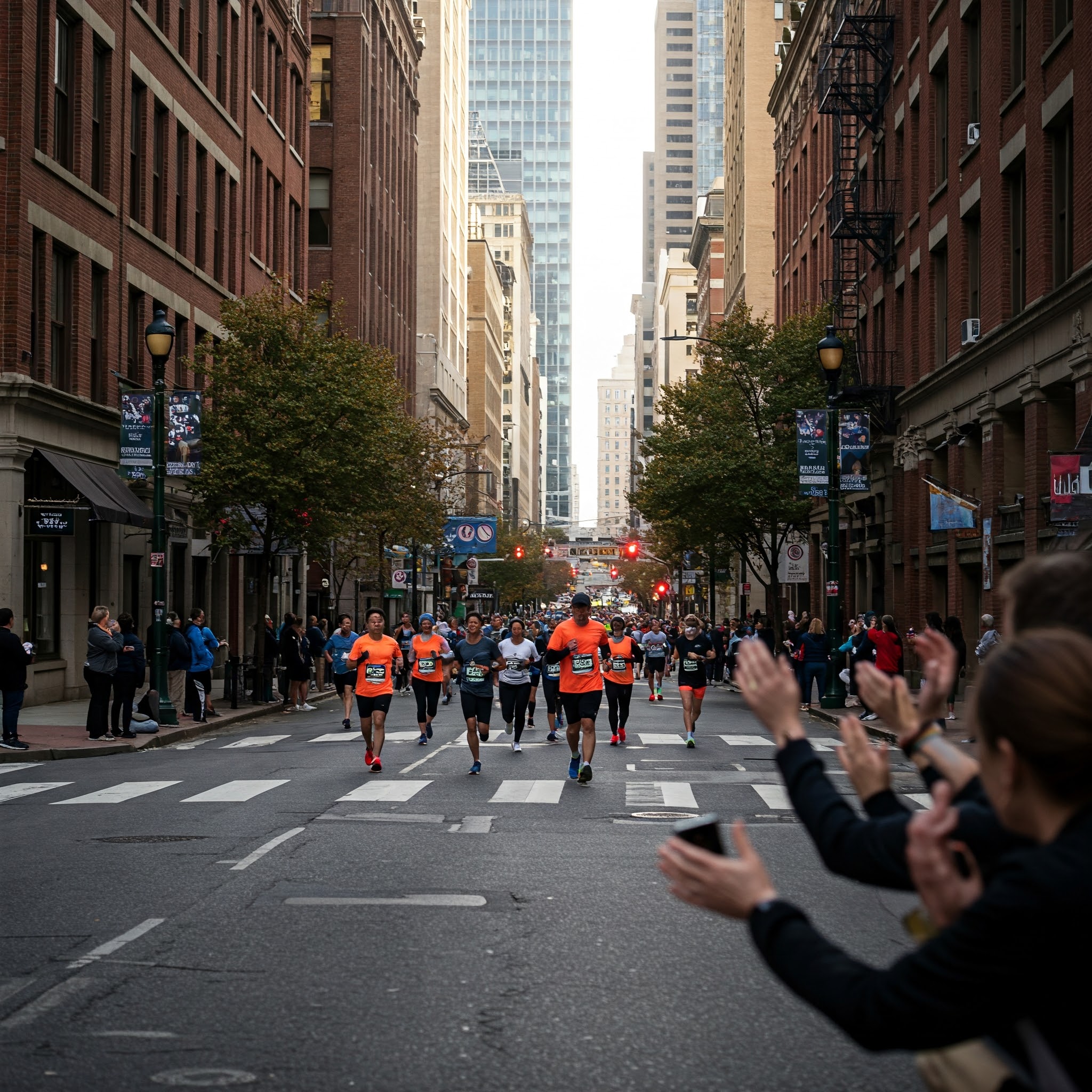

Supplement: S2 File — This file contains all images generated for the attribute levels described in Table 2. (ZIP) [file pone.0334308.s002.zip › S2_File/2-AUD 1.jpg]

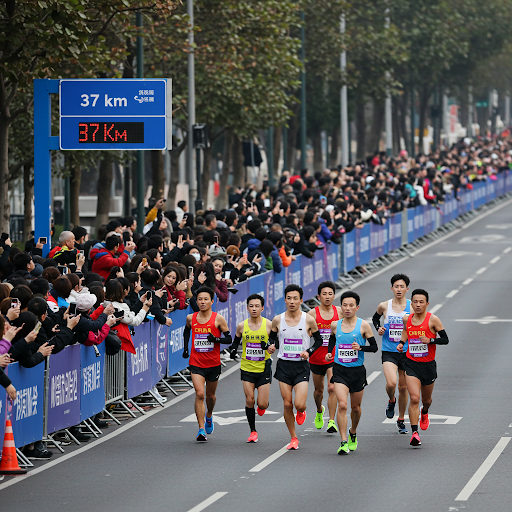

Supplement: S2 File — This file contains all images generated for the attribute levels described in Table 2. (ZIP) [file pone.0334308.s002.zip › S2_File/2-AUD 2.png]

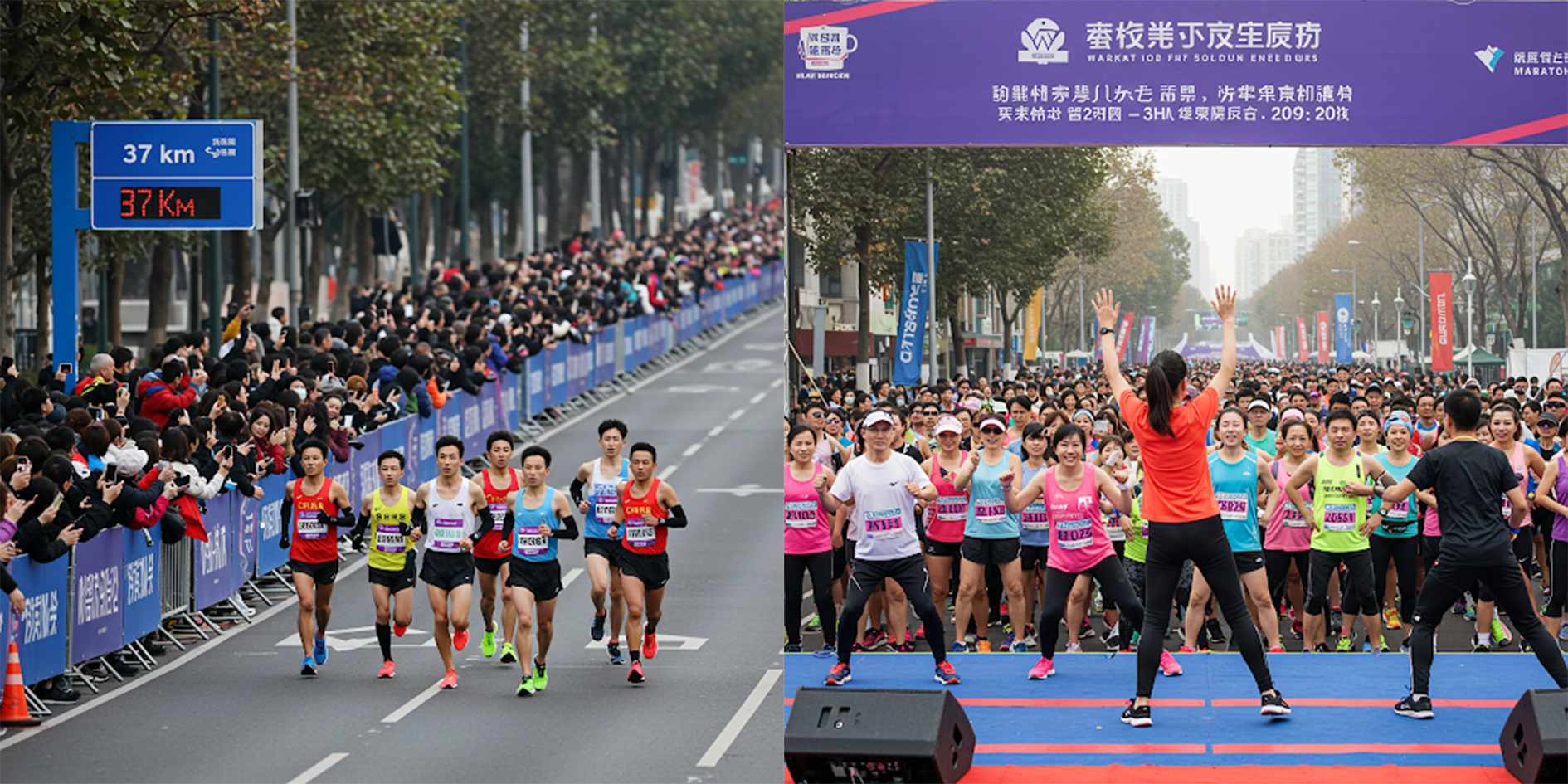

Supplement: S2 File — This file contains all images generated for the attribute levels described in Table 2. (ZIP) [file pone.0334308.s002.zip › S2_File/2-AUD 3.png]

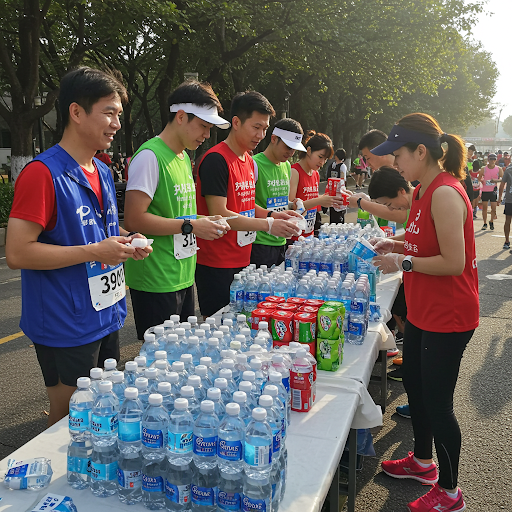

Supplement: S2 File — This file contains all images generated for the attribute levels described in Table 2. (ZIP) [file pone.0334308.s002.zip › S2_File/3-KIN 1.png]

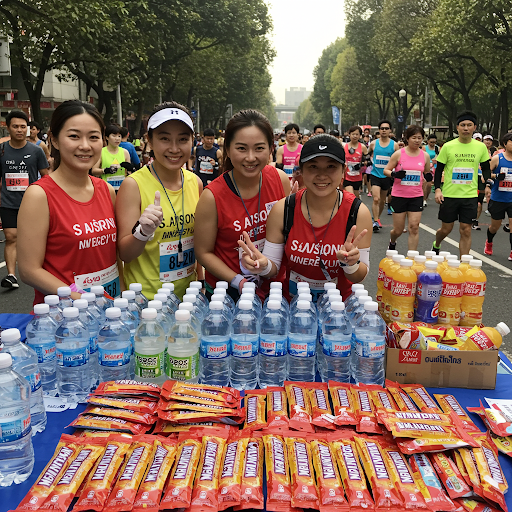

Supplement: S2 File — This file contains all images generated for the attribute levels described in Table 2. (ZIP) [file pone.0334308.s002.zip › S2_File/3-KIN 2.png]

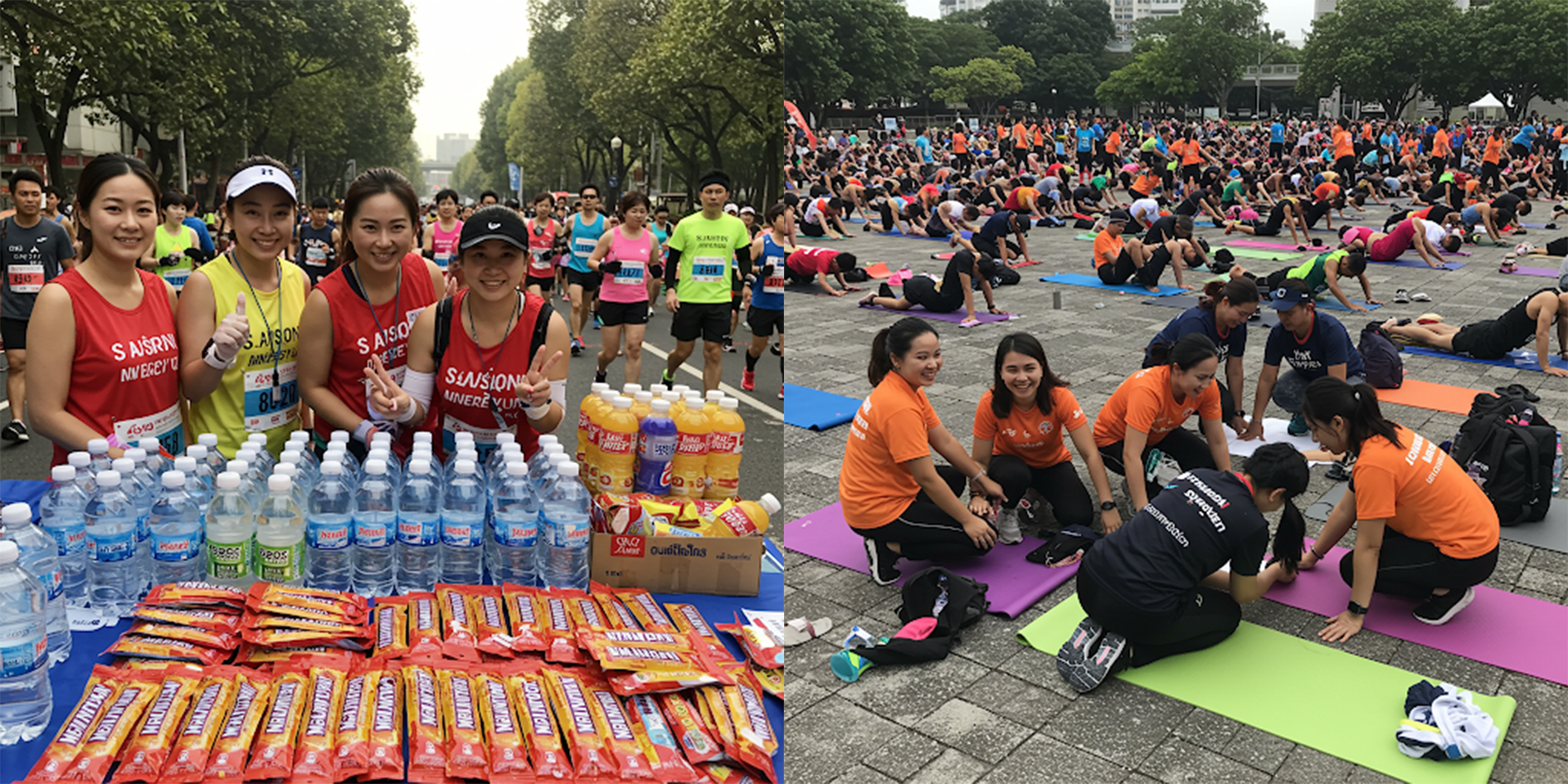

Supplement: S2 File — This file contains all images generated for the attribute levels described in Table 2. (ZIP) [file pone.0334308.s002.zip › S2_File/3-KIN 3.png]

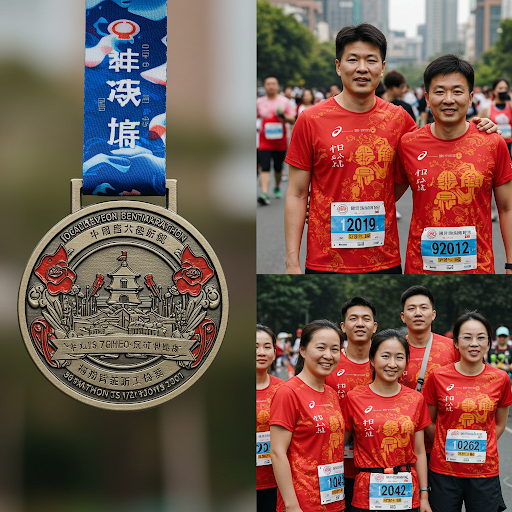

Supplement: S2 File — This file contains all images generated for the attribute levels described in Table 2. (ZIP) [file pone.0334308.s002.zip › S2_File/4-ERE 1.png]

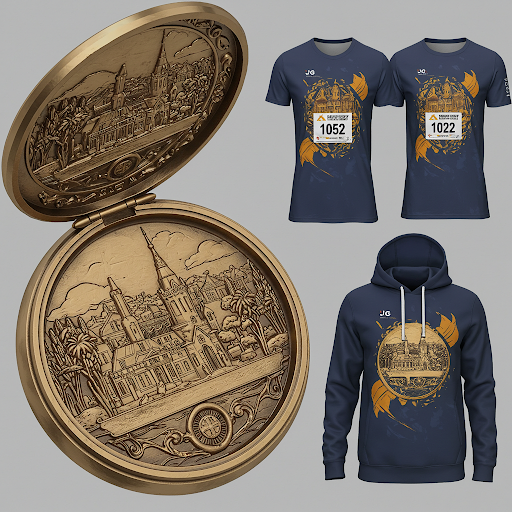

Supplement: S2 File — This file contains all images generated for the attribute levels described in Table 2. (ZIP) [file pone.0334308.s002.zip › S2_File/4-ERE 2.png]

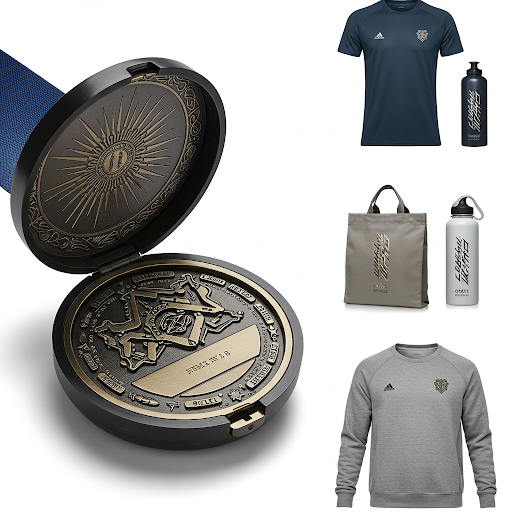

Supplement: S2 File — This file contains all images generated for the attribute levels described in Table 2. (ZIP) [file pone.0334308.s002.zip › S2_File/4-ERE 3.png]

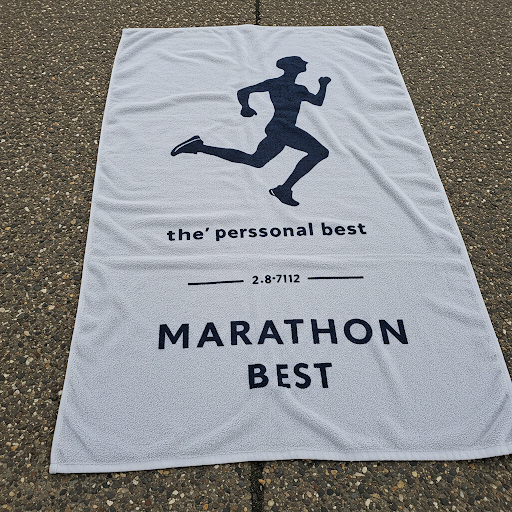

Supplement: S2 File — This file contains all images generated for the attribute levels described in Table 2. (ZIP) [file pone.0334308.s002.zip › S2_File/5-STR 1.png]

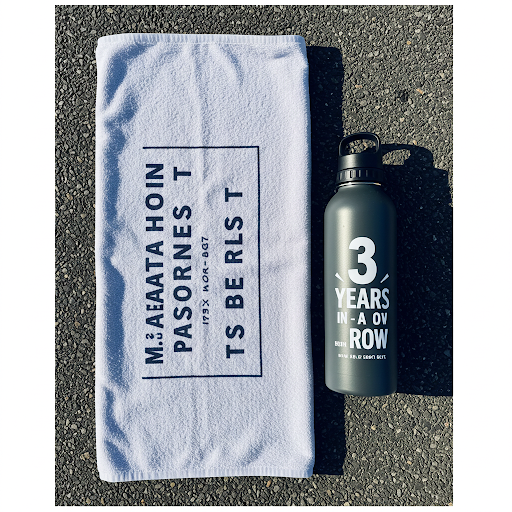

Supplement: S2 File — This file contains all images generated for the attribute levels described in Table 2. (ZIP) [file pone.0334308.s002.zip › S2_File/5-STR 2.png]

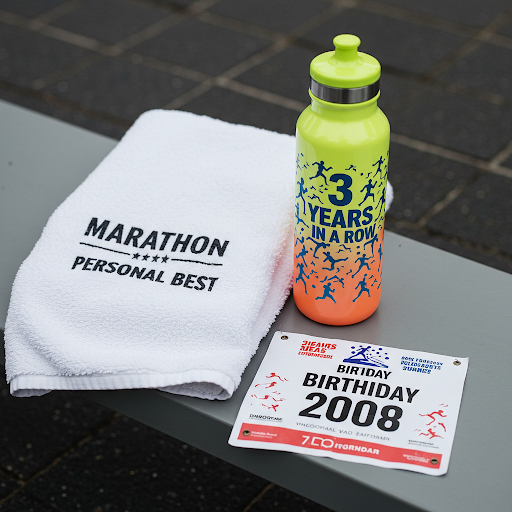

Supplement: S2 File — This file contains all images generated for the attribute levels described in Table 2. (ZIP) [file pone.0334308.s002.zip › S2_File/5-STR 3.png]

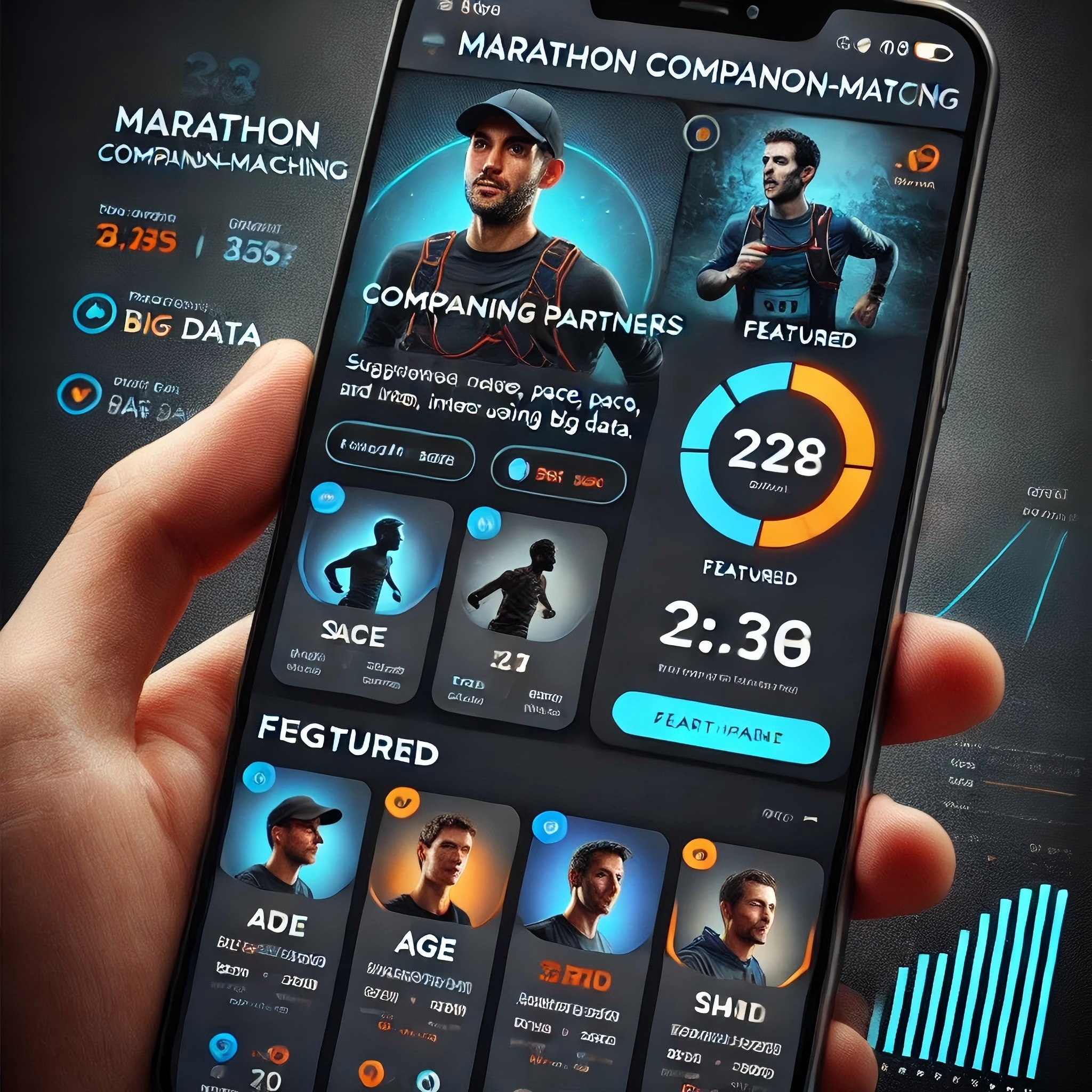

Supplement: S2 File — This file contains all images generated for the attribute levels described in Table 2. (ZIP) [file pone.0334308.s002.zip › S2_File/6-SBO 1.jpg]

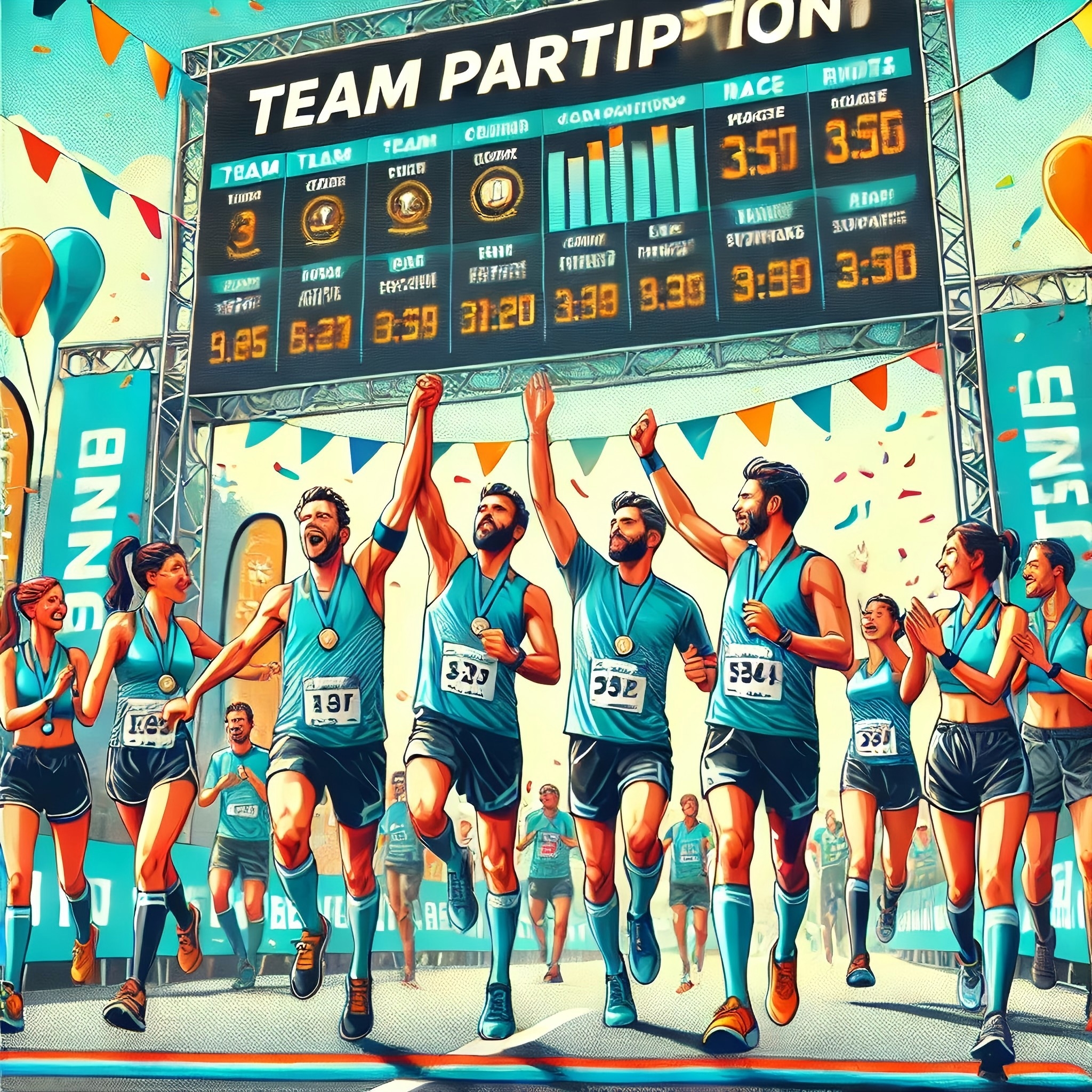

Supplement: S2 File — This file contains all images generated for the attribute levels described in Table 2. (ZIP) [file pone.0334308.s002.zip › S2_File/6-SBO 2.jpg]

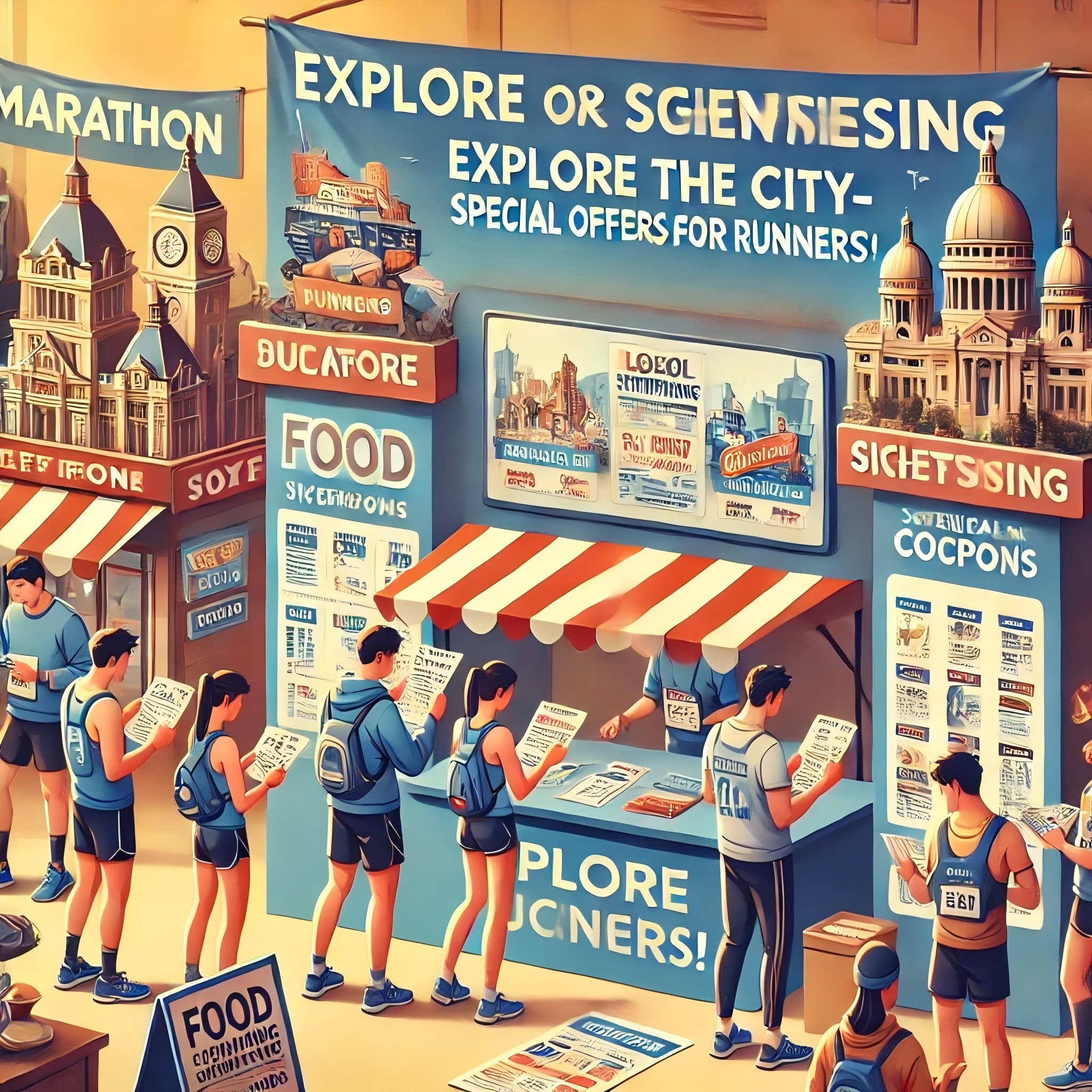

Supplement: S2 File — This file contains all images generated for the attribute levels described in Table 2. (ZIP) [file pone.0334308.s002.zip › S2_File/6-SBO 3.jpg]
